# Computational design of patterned interfaces using reduced order models

**DOI:** 10.1038/srep06231

**Published:** 2014-08-29

**Authors:** A. J. Vattré, N. Abdolrahim, K. Kolluri, M. J. Demkowicz

**Affiliations:** 1 CEA, DAM, DIF, F-91297 Arpajon, France; 2 MIT Department of Materials Science and Engineering, Cambridge MA, 02139

## Abstract

Patterning is a familiar approach for imparting novel functionalities to free surfaces. We extend the patterning paradigm to interfaces between crystalline solids. Many interfaces have non-uniform internal structures comprised of misfit dislocations, which in turn govern interface properties. We develop and validate a computational strategy for designing interfaces with controlled misfit dislocation patterns by tailoring interface crystallography and composition. Our approach relies on a novel method for predicting the internal structure of interfaces: rather than obtaining it from resource-intensive atomistic simulations, we compute it using an efficient reduced order model based on anisotropic elasticity theory. Moreover, our strategy incorporates interface synthesis as a constraint on the design process. As an illustration, we apply our approach to the design of interfaces with rapid, 1-D point defect diffusion. Patterned interfaces may be integrated into the microstructure of composite materials, markedly improving performance.

Composite materials with exceptional properties, such as radiation resistance^1^, extreme thermo-mechanical stability^2^, or high strength and ductility^3^, may be created by controlling the interfaces between composite constituents, in addition to the constituents themselves. One way to customize interface properties is by tailoring their internal structure. However, unlike free surfaces, which may be directly patterned^4^,^5^,^6^,^7^,^8^,^9^, interface structure is difficult to control because interfaces are buried within the material and cannot be directly accessed.

We propose an approach for computational design of patterned interfaces by tuning their crystallography and composition. We focus specifically on interfaces of the type illustrated in Fig. 1, whose internal structure contains coherent regions separated by inherent line defects, known as misfit dislocations^10^,^11^,^12^. Interface crystallography and composition are the natural “design space” for such interfaces because they govern the misfit dislocation pattern. This pattern, in turn, determines interface properties such as impurity precipitation^13^,^14^,^15^, point defect mobility^16^,^17^,^18^, and shearing resistance^19^,^20^. Thus, given the misfit dislocation pattern that yields a desired property, our goal is to predict the interface crystallography and composition that generate that pattern and—ideally—specify a synthesis route for that interface.

Misfit dislocation patterns may be predicted using atomic-level interface models^19^,^21^,^22^. We view this approach as a well-posed “forward problem.” By contrast, interface design—i.e., finding the interface that yields a desired misfit dislocation pattern—is an “inverse problem”^23^. In principle, it may be solved by repeatedly executing the forward problem over the design space. In practice, such a procedure is not feasible because of the high dimensionality of the design space (discussed below) and the substantial effort required for each forward problem solution.

Our approach to interface design is to construct a mesoscale (as opposed to atomic-level) model that predicts misfit dislocation patterns with accuracy comparable to atomistic simulations, but at a fraction of the cost. Our model is a reduced order model because it replaces the millions of variables associated with atomic positions with ≤15 variables needed to describe misfit dislocations. This reduced order mesoscale model, or ROMM, enables rapid scanning over the interface design space and thereby opens the path to computational design of patterned interfaces. To demonstrate its effectiveness, we use our ROMM to design interfaces that exhibit rapid, 1-D diffusion of point defects.

## Methods

### Design space

Solid-state interfaces exhibit limitless variety^12^,^24^,^25^. We focus on the subset of atomically sharp interfaces between metals whose crystal structures may be mapped into each other by uniform deformation gradients. Far from being restrictive, this subset defines a high-dimensional design space that includes all stress-free and uniformly strained face- and body-centered cubic (FCC, BCC) and tetragonal (FCT, BCT) metal pairs. Grain boundaries also fall within this subset. Extensions of our ROMM to other types of interfaces will be addressed in the Discussion section.

**Figure 1:**
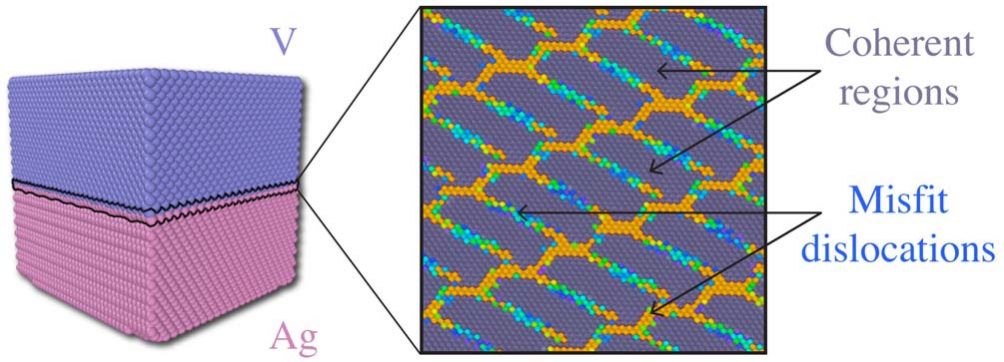
Ag terminal plane in a Ag/V interface (crystallography as in Results section with θ = 1°). Interface atoms are colored by their energy, showing coherent regions separated by misfit dislocations.

**Figure 2:**
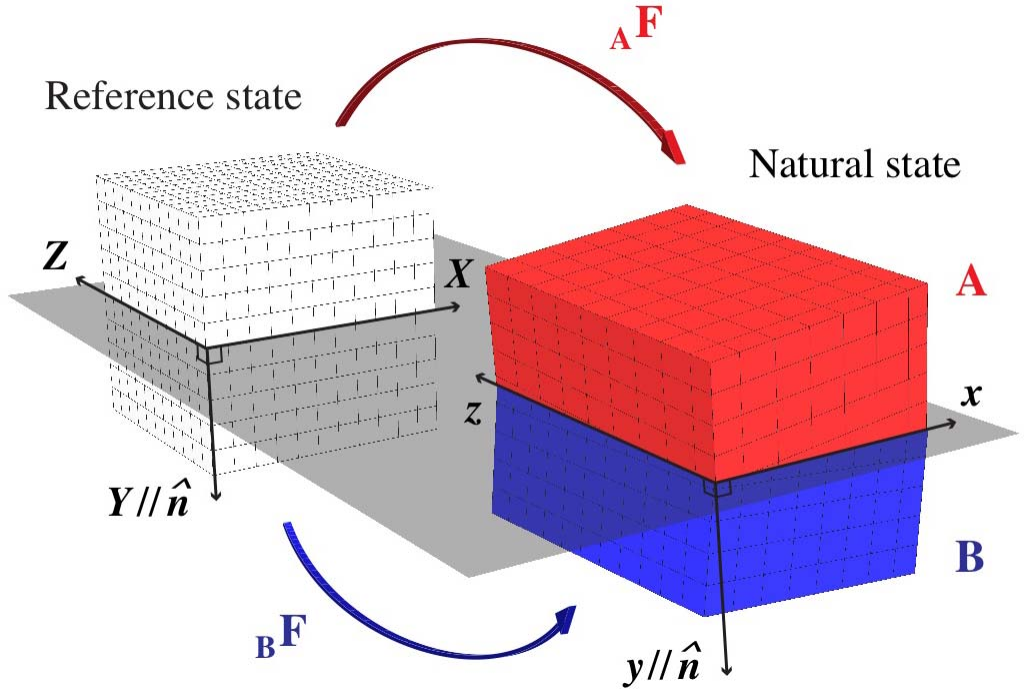
Interface crystallographic DOFs may be described with deformation gradients _A_F and _B_F, which map from a single crystal reference state to the natural state.

We describe an interface's crystallography by comparing it to a single crystal reference state^26^,^27^. Any interface of the type studied here may be described by two uniform deformation gradients: **_A_F** and **_B_F**, applied respectively to the parts of the reference crystal above (A) and below (B) the ***X-Z*** plane, as shown in Fig. 2. This mapping generates the “natural” interface state, with the interface along the ***x-z*** plane. **_A_F** and **_B_F** are chosen such that, in the natural state, parts A and B match the prescribed crystal structures and orientations of the solids joined at the interface. Misfit dislocation patterns, such as those in Fig. 1, are generated once the interface in its natural state is relaxed.

**_A_F** and **_B_F** may be represented as 3 × 3 matrices^28^,^29^. Together, they contain eighteen components. Six of them correspond to rotations of A and B. Since equal rotations of A and B along the interface plane do not affect interface structure, only five rotation components are needed to describe interface crystallography. The remaining twelve components of **_A_F** and **_B_F** describe strains. However, only the relative strain between A and B affects interface structure, reducing the number of independent strains to six. Therefore, there are in total eleven crystallographic degrees of freedom (DOFs) available for interface design. For grain boundaries, the strains are fixed, reducing the DOFs to the well-known five^12^.

In addition to crystallographic DOFs, interface structure also depends on the elastic properties of the adjoining materials^26^,^27^. A solid may have between two and twenty-one independent elastic constants, depending on its symmetry. Thus, a complete description of interface elasticity requires at least four elastic constants (if the adjoining materials are isotropic) and as many as forty-two. In practice, elastic constants depend on composition and crystal structure. Thus, rather than enumerate all their possible combinations, we simply refer to them as a potentially very large set of “compositional” DOFs available for interface patterning.

### State variables

**Figure 3:**
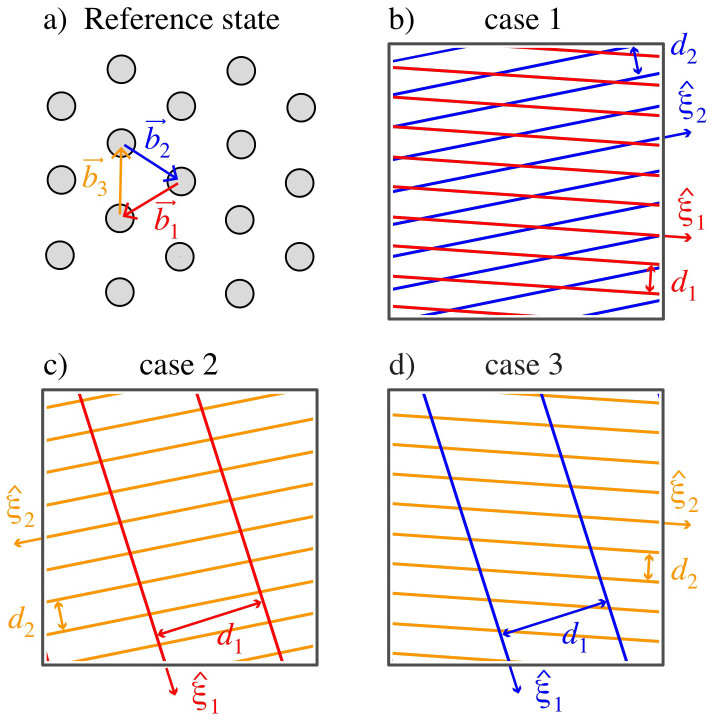
(a) Atom positions at the interface plane in the coherent reference state for a Cu/Nb interface with θ = 1°. (b) “Case 1” is a solution of the qFBE (Eqn. 1) that uses Burgers vectors 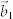 and 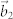 shown in (a). (b) “Case 2” uses 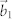 and 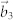 while (c) “case 3” uses 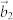 and 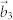. *d_i_* and 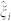 denote misfit dislocation spacings and directions.

Given the crystallographic and compositional DOFs of an interface, the resulting misfit dislocation pattern may be determined using atomistic simulations. Such simulations are costly because they track the positions of all atoms near the interface. It is far more efficient to describe interface structure in terms of the misfit dislocations themselves, instead of atoms. Misfit dislocation patterns for the interfaces of interest here consist of m sets of misfit dislocations. Each set contains an infinite number of straight, parallel, equally spaced dislocations, as illustrated in Fig. 3.b)–d). Thus, a minimal description of each set requires the dislocations' line direction 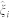, their spacing *d_i_*, and Burgers vector 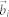 (i = 1…m).


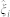
 is a unit vector in the interface plane and so may be specified using a single variable while *d_i_* and 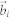 require one and three variables, respectively. Therefore, as few as 5m state variables are required to describe all m sets of dislocations, as opposed to the millions of variables needed to specify locations of all atoms near an interface. Potential additional state variables will be considered in the Discussion section.

### Reduced order mesoscale model (ROMM)

We construct a ROMM that predicts, without recourse to atomistic simulations, the 5m state variables of misfit dislocation patterns from the 15+ DOFs describing interface crystallography and composition. The crystallographic DOFs and state variables are related by the quantized Frank-Bilby equation (qFBE)^12^,^30^,^31^,^32^: 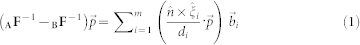
The left hand side is derived from a Burgers circuit enclosing a probe vector 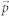 that lies in the interface plane. It expresses the sum of misfit dislocation Burgers vectors crossed by 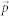 (averaged over all possible origins of 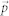) from deformation gradients **_A_F** and **_B_F**. The right hand side of the qFBE writes out this sum in terms of up to m = 3 sets of misfit dislocations.

Solutions for 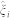 and *d_i_* may be obtained from the qFBE using O-lattice theory^29^,^32^,^33^. The determination of 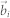 involves a subtle problem in anisotropic elasticity^34^, which we recently solved^27^. However, these two steps do not yield a unique solution. Rather, they provide as many candidate solutions as there are combinations of linearly independent Burgers vectors in the reference state^29^,^35^. We determine which of these candidate solutions is the correct one by calculating their interface energies, γ, and choosing the one with lowest γ.

Misfit dislocations may be viewed as Volterra dislocations that have been inserted into the coherent reference state^27^, suggesting that γ be expressed as 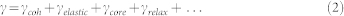
γ_coh_ is the interface energy of the coherent reference state and the remaining terms are due to misfit dislocations: elastic strain energy γ_elastic_, core energy γ_core_, energy due to relaxations of the misfit dislocation network γ_relax_, and perhaps additional terms that have not been identified.

For our purposes, it is not necessary to calculate the absolute value of γ, but rather only differences in γ between the candidate solutions of the qFBE. γ_coh_ does not contribute to these differences because each solution represents different Volterra dislocations inserted into the same coherent reference state. We further make the hypothesis—to be validated later—that core and relaxation energies are not the main contributors to differences in γ. We therefore concentrate on computing the elastic energy contribution: γ_elastic_.

After enumerating all the solutions to the qFBE (Eqn. 1), we compute the complete elastic fields for each one, as described in Ref. 27. These fields satisfy four boundary conditions: (i) absence of net far-field strains, (ii) consistency of far-field rotations with the misorientation prescribed in **_A_F** and **_B_F**, (iii) no net tractions along the interface, and (iv) a position-dependent displacement discontinuity, i.e. “disregistry”^36^, across the interface consistent with the misfit dislocation pattern of interest. Disregistries may be compared directly with atomistic simulations^19^,^20^,^37^,^38^. Our calculation is fully anisotropic and may be performed for any number of independent elastic constants. It is therefore capable of accounting for all compositional DOFs under consideration here.

When all boundary conditions are satisfied, the interface is free of far-field stresses. However, short-range stresses persist near the interface. These short-range fields are used to compute interface elastic energies: 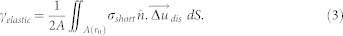
*A* is the domain of integration: a single unit cell of the misfit dislocation pattern. To eliminate the unphysical divergence of stresses at dislocation cores, A excludes the region within a cutoff distance 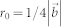 of the dislocation cores. 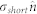 and 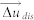 are the traction and disregistry vectors at the interface, respectively. By applying Eqn. 3 to all candidate solutions of the qFBE (Eqn. 1), we predict which one has lowest γ_elastic_ and therefore describes the likeliest misfit dislocation pattern.

## Results

### Validation

Our ROMM is a significant simplification of a complex problem and requires validation. To this end, we compare the output of our ROMM with atomistic calculations. We focus on heterophase interfaces between {111} planes of FCC metals and {110} planes of BCC metals. Such interfaces contain misfit dislocations of unlike character and asymmetric arrangement^19^,^22^,^28^,^34^. Thus, they provide an opportunity for rigorous validation of our ROMM. They are also convenient for atomistic simulations because embedded atom method (EAM)^39^ potentials are available for several FCC/BCC binaries.

We validate our ROMM against four interface compositions: Cu/Nb^40^, Ag/V^41^, Cu/Fe^42^, and Cu/Mo^43^. These choices fix the elastic constants, crystal structures, and lattice parameters of the adjoining constituents. Because we restrict attention to interfaces along FCC {111} and BCC {110} planes, only one crystallographic DOF remains to be specified: the twist angle θ describing the relative rotation of the crystals parallel to the interface plane.

We measure θ with respect to the Nishiyama-Wasserman orientation relation, where a BCC <100> direction is parallel to a FCC <110> direction (see supplementary note)^44^,^45^. 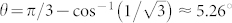 yields the Kurdjumov-Sachs orientation relation (KS OR)^46^. Due to the symmetry of the interface planes, all crystallographically distinct interfaces fall within 0° ≤ θ ≤ 15°. However, we limit our analysis to 0° ≤ θ ≤ 10° because for greater twists, misfit dislocations are too closely spaced to characterize reliably in atomic models.

For any composition and θ, the qFBE has three distinct candidate solutions^29^. Each consists of two sets of misfit dislocations (m = 2) and corresponds to one of three combinations of interfacial Burgers vectors, defined in the coherent reference state. For example, Fig. 3.a) shows the Burgers vectors in the reference state of a Cu/Nb interface with θ = 1°. The first candidate, termed “case 1,” uses Burgers vectors 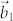 and 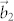. “Case 2” and “case 3” use Burgers vectors 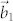, 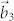 and 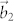, 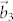, respectively. Each case yields a different misfit dislocation pattern, as shown in Fig. 3.b) –d). The coherent reference state is the same for all three cases. **_A_F** and **_B_F** are chosen such that 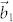, 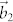, and 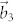 map to 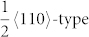 FCC directions, 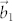 maps to a <100>-type BCC direction, and 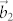, and 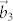 map to 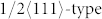 BCC directions.

Using our ROMM, we compute γ_elastic_ of all three cases for each composition and θ of interest. For all interfaces, we also construct atomic-scale models by joining cylindrical FCC and BCC blocks following the required interface crystallography. The models are large enough to contain a representative area of the misfit dislocation pattern and to avoid elastic images from free surfaces. The models are relaxed using EAM potentials^40^,^41^,^42^,^43^ and analyzed to extract interface energies and misfit dislocation structures (see supplementary note). All ROMM calculations use lattice parameters and elastic constants predicted by these EAM potentials.

**Figure 4:**
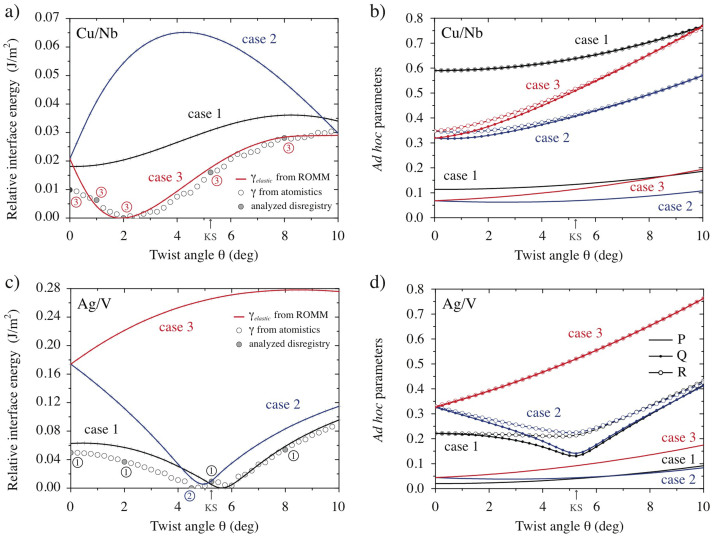
Interface energies computed as a function of θ using our ROMM and atomistic modeling for (a) Cu/Nb and (c) Ag/V. Filled circles indicate atomic models whose disregistry was analyzed. The ringed numbers next to them state the case that best matches the atomic disregistry. *Ad hoc* parameters P, Q, and R (Eqn. 4, 5, and 6) for (b) Cu/Nb and d) Ag/V.

Fig. 4.a) compares γ_elastic_ from our ROMM with γ from atomistic simulations for Cu/Nb interfaces. Since we are only interested in the relative energies of the three cases, both the ROMM and atomistic data are shifted so that their energy minima occur at 0 J/m^2^. The ROMM predicts that case 3 has lowest γ_elastic_ for all θ. Furthermore, γ_elastic_ for case 3 is in near perfect quantitative agreement with γ for θ>1°.

Fig. 4.c) shows a similar comparison for Ag/V interfaces. Here, the ROMM predicts that case 1 has lowest γ_elastic_ for all θ outside 4.25°<θ<5.25°, where γ_elastic_ is lowest for case 2. γ_elastic_ and γ are in qualitative agreement over the entire twist angle range and in quantitative agreement for θ>5°. As detailed in the supplementary note, we find comparable agreement between the ROMM and atomistic interface energies for the remaining two compositions we studied.

Agreement between γ_elastic_ and γ is not sufficient to validate our ROMM. For that, we must determine whether the lowest energy cases predicted by the ROMM match the misfit dislocation patterns in our atomistic simulations. To this end, we compare the disregistries of the two models (see supplementary note). Each of the three qFBE solutions predicts a different misfit dislocation pattern and therefore also a different disregistry. Our goal is to compare the disregistries of all three cases with that found in atomistic simulations. Our ROMM is validated if the case with lowest γ_elastic_ has the best match with the atomistic disregistry.

As shown in Fig. 4.a) and c), the disregistry analysis is in agreement with our ROMM predictions for all Cu/Nb and Ag/V interfaces except Cu/Nb at θ = 0°. We attribute the disagreement to the reconstruction of the misfit dislocation network that is known to occur at that interface^22^. One further case of disagreement where dislocation network reconstruction occurs is found for Cu/Mo at θ = 0° (see supplementary note). However, the agreement between our ROMM and the atomistic models is excellent, overall.

Our ROMM may be compared with several *ad hoc* parameters proposed previously to determine which of the cases predicted by the qFBE is likeliest. Bollmann suggested that the likeliest case minimizes^29^

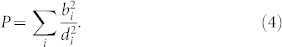
The rationale for this parameter is analogous to the “Frank rule” for predicting dislocation reactions^36^. Similarly, Ecob and Ralph propose to distinguish between cases using parameters^35^

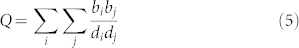
or 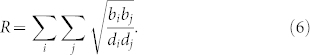
Fig. 4.b) and d) plot these parameters for Cu/Nb and Ag/V interfaces. Comparing with Fig. 4.a) and c), we see that none of them predicts the misfit dislocation patterns seen in atomistic models. For example, for Cu/Nb, all three parameters favor case 2, while the true interface structure is case 3. We therefore view our ROMM as superior to these parameters and as validated for the purpose of computational design of patterned interfaces.

### Application to interface patterning

A validated ROMM for predicting misfit dislocation patterns may be used for computational interface design. Potential applications include interface patterning for templated precipitation of impurities^13^,^14^,^15^, tailored shearing resistance^20^, or twin nucleation^47^. As a demonstration, we apply our ROMM to the design of interfaces with fast, 1-D point defect diffusion.

Previously, we detailed a distinctive mechanism of point defect diffusion at a Cu/Nb interface^16^,^17^,^18^. In it, interfacial vacancies or interstitials undergo rapid migration parallel to one set of misfit dislocations and considerably slower migration perpendicular to it. This mechanism may also operate at other interfaces provided that their misfit dislocation pattern meets two criteria. First, it must contain one set of misfit dislocations of nearly perfect screw character and another of non-screw character. Second, the distance between misfit dislocation intersections along the fast migration direction must be small, i.e. below ~2 nm.

We use our ROMM to design FCC/BCC interfaces that meet these two criteria and therefore exhibit rapid 1-D point defect diffusion. We consider interfaces of the type described in Validation subsection, but rather than restricting ourselves to pure elements, we use alloys of Cu and Ni for the FCC side and alloys of Nb and V for the BCC side. By changing the alloy composition, we may continuously vary the lattice parameters and elastic constants of the adjacent crystals. We assume that all of these properties follow Vegard's law, i.e. that they may be interpolated linearly in composition between the single-component properties.

For example, the lattice parameter of the Cu-Ni alloy may be expressed as 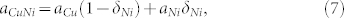
where *δ_Ni_* is the atomic fraction of Ni in the alloy. Thus, *δ_Ni_* is the only compositional DOF for the FCC component. Similarly, the atomic fraction of V, *δ_V_*, is the only compositional DOF for the BCC component. Replacing Vegard's law with a nonlinear composition-property relation would not increase the number of compositional DOFs and so would not change the interface design process, although it might alter its outcome. The only crystallographic DOF we will consider is the twist angle θ.

**Figure 5:**
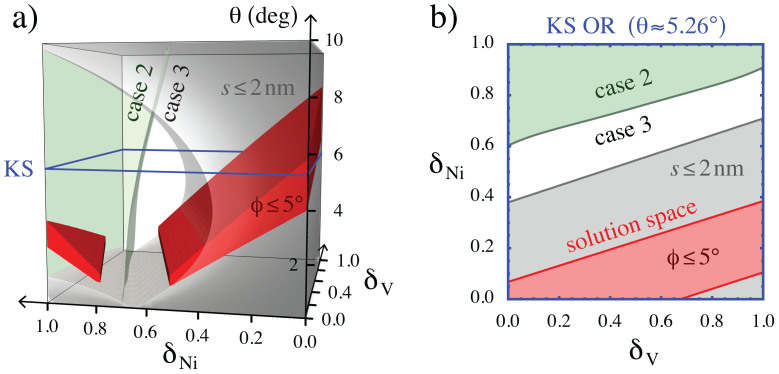
(a) The 3-D δ_Ni_-δ_V_-θ design space and (b) a planar section through it for θ≈5.26°. Domains corresponding to case 2 and case 3 are delimited by a 2-D surface in (a). The red, airfoil-shaped envelopes labeled “ϕ ≤ 5°” enclose interfaces that have one set of nearly screw misfit dislocations as well as one non-screw set. The shaded region labeled “s ≤ 2 nm” encloses interfaces with misfit dislocation intersections less than 2 nm apart along the set of misfit dislocations with screw character. The solution space for interfaces with rapid 1-D point defect diffusion is the intersection between this region and the red airfoils.

Using our ROMM, we find that the *δ_Ni_−δ_V_−θ* design space contains regions with case 2 and case 3 misfit dislocation patters, as shown in Fig. 5.a). With this information, we plot envelopes where the criteria for rapid 1-D diffusion are met. Inside the red, airfoil-shaped regions in Fig. 5.a), one set of dislocations is within 5° of perfect screw character while the other set of dislocations is non-screw. Within the region shaded gray, the spacing between misfit dislocation intersections along the screw set of dislocations is smaller than 2 nm. The intersection between the red airfoils and the gray shaded region represents misfit dislocation patterns that meet the criteria for rapid, 1-D diffusion.

Ideally, a design for a patterned interface identifies not only crystallographic and compositional DOFs, but also provides a synthesis route for that interface. In this example, we may incorporate synthesis as an additional constraint on the design process. For example, interfaces between FCC {111} and BCC {110} planes may be made using physical vapor deposition (PVD)^48^. PVD gives interfaces in the KS OR, where θ≈5.26°. Thus, the design envelope with a PVD synthesis route is obtained by restricting the 3-D design space to a planar submanifold with θ≈5.26°, shown in Fig. 5.b). Other interface processing methods, such as molecular beam epitaxy (MBE)^49^, diffusion bonding^50^, precipitation^51^, or severe plastic deformation (SPD)^52^, may be represented by different constraints on the design space.

In principle, the interface design process described above may be carried out using atomistic modeling. In practice, the resources required would be prohibitive. Nearly 69 thousand distinct interfaces were examined to create the 3-D map in Fig. 5.a). Assuming an interatomic potential is available, we estimate 5 person-hours and 24 CPU hours to compute one interface structure using atomistic modeling. If a potential is not available, then considerable additional human effort is required to create one. Alternatively, much greater computing resources may be used to carry out first principles atomistic simulations. Thus, constructing the map in Fig. 5.a) using atomistic modeling is infeasible. By contrast, <7 CPU minutes are required to compute an interface structure using our ROMM. Furthermore, since the ROMM takes DOFs such as elastic constants as direct inputs, it may be applied to a wide range of compositions without further effort.

## Discussion

The promise of using designer solid-state interfaces to impart unprecedented new functionalities to materials has been widely recognized^1^,^2^,^3^,^14^. We presented a strategy for rapid computational design of interfaces with tailored misfit dislocation patterns. Computational design of patterned interfaces may be viewed as an inverse problem where the DOFs that yield an interface with prescribed state variables are found. Our strategy for solving such inverse problems relies on a reduced order mesoscale model, or ROMM, for predicting interface state variables from interface DOFs. We validated our ROMM through direct comparisons with atomistic models and applied it to an illustrative interface design problem. Synthesis routes may be incorporated as constraints in the interface design process.

We illustrated our ROMM-based strategy on the example of interface design for fast, 1-D point defect diffusion. A critical piece of knowledge that enabled that design was the quantitative description of the mechanism of point defect diffusion at Cu/Nb interfaces, which was already available^16^,^17^,^18^. Such a model linking misfit dislocation patterns to interface properties may itself be viewed as a kind of ROMM. Further applications of interface design will also require quantitative models linking interface structure to interface properties. Several are already available. For example, interfacial precipitates wet high-energy regions near misfit dislocations while avoiding low-energy, non-wetting coherent patches^13^. Thus, control of interface structure may be used for templated precipitation^15^, in analogy to free surfaces patterned to control water nucleation^5^ or to enable self-cleaning^7^. Similarly, impurity trapping^53^,^54^ and shear resistance^20^ differ for coherent patches and misfit dislocations, opening a path to controlling interfacial segregation and mechanical properties.

Although motivated by computational design of patterned interfaces, the ROMM described here may also find applications in predicting phase transformations or microstructure evolution. These processes depend on interface structure and energy^55^,^56^, both of which may be computed using our ROMM. For example, Cu/Nb interface energy is minimized at θ≈2° in Fig. 4.a). Thus, given a Cu/Nb interface initially at some other θ and assuming no constraints from surrounding grains, we expect the adjoining crystals to rotate until the minimum energy misorientation is reached. This reasoning may be extended to predict orientations of martensite habit planes^57^ and grain boundary energies in polycrystals^58^.

Our ROMM distinguishes between candidate interface structures predicted by the qFBE based on strain energies γ_elastic_ calculated using anisotropic elasticity theory^26^,^27^. This is the underlying reason for the better performance of our ROMM compared to previously proposed parameters^29^,^35^. It is nevertheless remarkable that γ_elastic_ is often in quantitative agreement with γ computed using atomistics. The true interface energy, γ, involves several terms besides γ_elastic_, e.g. contributions from dislocation network relaxations and from dislocation core energies. Indeed, the former appear to play a role when the agreement between γ_elastic_ and γ is not perfect. For example, the misfit dislocation network in Cu/Nb at θ = 0° is known to reconstruct into a hexagonal pattern^22^, explaining why γ<γ_elastic_ in Fig. 4.a). We have found evidence for similar reconstructions in Ag/V for θ<4.5°.

More surprisingly, the agreement between γ_elastic_ and γ is very good despite the low spacing between misfit dislocations in the FCC/BCC interfaces in the Results section: in some cases, it is comparable to the dislocation core size, i.e. <1 nm. The fact that γ_elastic_ nevertheless provides a good estimate for γ is likely not because dislocation core contributions γ_core_ may be neglected, but rather because they are related to the elastic energy contribution. Such a relationship may be rationalized through the Peierls-Nabarro model^59^,^60^ or analogous arguments^61^,^62^.

Numerous extensions of the ROMM described here may be envisioned, for example to interfaces with three sets of misfit dislocations, interfaces whose reference states are not single crystals (e.g. grain boundaries with small misorientations from coherent states), or interfaces between crystals of finite size (to account for free surfaces or layered morphologies). By increasing the number of state variables, the ROMM may be extended to account for misfit dislocation network relaxations, dislocation core structures, solute atmospheres, or non-equilibrium interface states.

Expanding the subset of interfaces treated by the ROMM would require increasing the number of DOFs it takes as inputs. For example, modeling interfaces between crystals that cannot be mapped to each other using uniform deformation gradients alone may require new DOFs for sublattice atom shuffles. Additional DOFs may be required to describe non-elastic energy contributions, e.g. electrostatic ones in interfaces between ionic solids. Line defects other than dislocations may be important for some interfaces^57^,^63^.

Since the time of Read and Shockley^64^, it has been argued that misfit dislocation models of interfaces must break down when the distance between dislocations becomes small. However, in view of the numerous possible improvements and extensions mentioned above, it is not clear at what dislocation spacing this breakdown would occur or how far it may be pushed back. As ROMMs for interface structure become more complex, determining the limits of their validity may increasingly require recourse to formal uncertainty quantification (UQ) methods, such as ones based on Bayesian inference^65^.

## Author Contributions

A.J.V. created figures 2-5 and S2 as well as table SI, N.A. created figure 1 and S3 as well as table SII, K.K. created figure S1 and wrote the first section of the supplementary note, M.J.D. wrote the manuscript text and the second section of the supplementary note. All authors reviewed the manuscript.

## Supplementary Material

Supplementary Information Supplementary Note

## References

[b1] Han W. Z. *et al.* Design of Radiation Tolerant Materials Via Interface Engineering. Adv Mater 25, 6975–6979 (2013). 24352985 10.1002/adma.201303400

[b2] Zheng S. J. *et al.* High-strength and thermally stable bulk nanolayered composites due to twin-induced interfaces. Nat Commun 4, 1696 (2013). 23591863 10.1038/ncomms2651

[b3] Zhu T., Li J., Samanta A., Kim H. G. & Suresh S. Interfacial plasticity governs strain rate sensitivity and ductility in nanostructured metals. Proc Natl Acad Sci U.S.A. 104, 3031–3036 (2007). 17360604 10.1073/pnas.0611097104 PMC1805608

[b4] Sinitskii A. & Tour J. M. Patterning Graphene through the Self-Assembled Templates: Toward Periodic Two-Dimensional Graphene Nanostructures with Semiconductor Properties. J Am Chem Soc 132, 14730–14732 (2010). 20886879 10.1021/ja105426h

[b5] Varanasi K. K., Hsu M., Bhate N., Yang W. S. & Deng T. Spatial control in the heterogeneous nucleation of water. Appl Phys Lett 95, 094101 (2009).

[b6] Chan W. L. & Chason E. Making waves: Kinetic processes controlling surface evolution during low energy ion sputtering. J Appl Phys 101, 121301 (2007).

[b7] Blossey R. Self-cleaning surfaces - virtual realities. Nat Mater 2, 301–306 (2003). 12728235 10.1038/nmat856

[b8] Aizenberg J., Black A. J. & Whitesides G. M. Control of crystal nucleation by patterned self-assembled monolayers. Nature 398, 495–498 (1999).

[b9] Gupta V. K. & Abbott N. L. Design of surfaces for patterned alignment of liquid crystals on planar and curved substrates. Science 276, 1533–1536 (1997).

[b10] Amelinckx S. The direct observation of dislocations (Academic Press, 1964).

[b11] Laird C. & Aaronson H. I. Dislocation structures of broad faces of Widmanstätten γ plates in an Al-15% Ag alloy. Acta Metall 15, 73 (1967).

[b12] Sutton A. P. & Balluffi R. W. Interfaces in Crystalline Materials (Oxford University Press, 1995).

[b13] Kashinath A., Misra A. & Demkowicz M. J. Stable Storage of Helium in Nanoscale Platelets at Semicoherent Interfaces. Phys Rev Lett 110, 086101 (2013). 23473167 10.1103/PhysRevLett.110.086101

[b14] Demkowicz M. J., Misra A. & Caro A. The role of interface structure in controlling high helium concentrations. Curr Opin Solid State Mat Sci 16, 101–108 (2012).

[b15] Hetherly J., Martinez E., Di Z. F., Nastasi M. & Caro A. Helium bubble precipitation at dislocation networks. Scr Mater 66, 17–20 (2012).

[b16] Kolluri K. & Demkowicz M. J. Formation, migration, and clustering of delocalized vacancies and interstitials at a solid-state semicoherent interface. Phys Rev B 85 (2012).

[b17] Kolluri K. & Demkowicz M. J. Dislocation mechanism of interface point defect migration. Phys Rev B 82, 193404 (2010).

[b18] Kolluri K., Demkowicz M. J., Hoagland R. G. & Liu X. Y. Behavior of Vacancies and Interstitials at Semicoherent Interfaces. JOM 65, 374–381 (2013).

[b19] Demkowicz M. J., Wang J. & Hoagland R. G. Interfaces between dissimilar crystalline solids. Dislocations in Solids Hirth, J. P. (ed.) 141–205 (Elsevier, 2008).

[b20] Demkowicz M. J. & Thilly L. Structure, shear resistance, and interaction with point defects of interfaces in Cu-Nb nanocomposites synthesized by severe plastic deformation. Acta Mater 59, 7744 (2011).

[b21] Wang J., Zhang R., Zhou C., Beyerlein I. J. & Misra A. Characterizing interface dislocations by atomically informed Frank-Bilby theory. J Mater Res 28, 1646–1657 (2013).

[b22] Wang J., Zhang R. F., Zhou C. Z., Beyerlein I. J. & Misra A. Interface dislocation patterns and dislocation nucleation in face-centered-cubic and body-centered-cubic bicrystal interfaces. Int J Plast 53, 40–55 (2014).

[b23] Tarantola A. Inverse problem theory and methods for model parameter estimation (Society for Industrial and Applied Mathematics, 2005).

[b24] Mishin Y., Asta M. & Li J. Atomistic modeling of interfaces and their impact on microstructure and properties. Acta Mater 58, 1117–1151 (2010).

[b25] Cantwell P. R., Tang M., Dillon S. J., Luo J., Rohrer G. S. & Harmer M. P. Grain boundary complexions. Acta Mater 62, 1–48 (2014).

[b26] Vattré A. J. & Demkowicz M. J. Effect of interface dislocation Burgers vectors on elastic fields in anisotropic bicrystals. Comput Mater Sci 88, 110–115 (2014).

[b27] Vattré A. J. & Demkowicz M. J. Determining the Burgers vectors and elastic strain energies of interface dislocation arrays using anisotropic elasticity theory. Acta Mater 61, 5172–5187 (2013).

[b28] Bollmann W. O-lattice calculation of an fcc-bcc interface. Phys Status Solidi A 21, 543–550 (1974).

[b29] Bollmann W. Crystal defects and crystalline interfaces (Springer-Verlag, 1970).

[b30] Frank F. C. The resultant content of dislocations in an arbitrary intercrystalline boundary. A symposium on the plastic deformation of crystalline solids (Carnegie Institute of Technology and Office of Naval Research, 1950).

[b31] Bilby B. A., Bullough R. & Smith E. Continuous distributions of dislocations: a new application of the methods of non-Riemannian geometry. Proc R Soc A 231, 263–273 (1955).

[b32] Yang J. B., Nagai Y., Yang Z. G. & Hasegawa M. Quantization of the Frank-Bilby equation for misfit dislocation arrays in interfaces. Acta Mater 57, 4874–4881 (2009).

[b33] Knowles K. M. The dislocation geometry of interphase boundaries. Philos Mag A 46, 951–969 (1982).

[b34] Hirth J. P., Pond R. C., Hoagland R. G., Liu X. Y. & Wang J. Interface defects, reference spaces and the Frank-Bilby equation. Prog Mater Sci 58, 749–823 (2013).

[b35] Ecob R. C. & Ralph B. Geometrical model for the energy of semicoherent interphase interfaces. Proc Natl Acad Sci U.S.A. 77, 1749–1753 (1980). 16592796 10.1073/pnas.77.4.1749 PMC348582

[b36] Hirth J. P. & Lothe J. Theory of Dislocations (Wiley, 1982).

[b37] Hoagland R. G., Mitchell T. E., Hirth J. P. & Kung H. On the strengthening effects of interfaces in multilayer fcc metallic composites. Philos Mag A 82, 643–664 (2002).

[b38] Wang J. & Misra A. An overview of interface-dominated deformation mechanisms in metallic multilayers. Curr Opin Solid State Mat Sci 15, 20–28 (2011).

[b39] Daw M. S. & Baskes M. I. Embedded-Atom Method - Derivation And Application To Impurities, Surfaces, And Other Defects In Metals. Phys Rev B 29, 6443–6453 (1984).

[b40] Demkowicz M. J. & Hoagland R. G. Simulations of collision cascades in Cu-Nb layered composites using an EAM interatomic potential. Int J Appl Mech 1, 421 (2009).

[b41] Wei Q. M., Liu X. Y. & Misra A. Observation of continuous and reversible bcc-fcc phase transformation in Ag/V multilayers. Appl Phys Lett 98, 111907 (2011).

[b42] Ludwig M., Farkas D., Pedraza D. & Schmauder S. Embedded atom potential for Fe-Cu interactions and simulations of precipitate-matrix interfaces. Model Simul Mater Sci Eng 6, 19–28 (1998).

[b43] Gong H. R., Kong L. T. & Liu B. X. Metastability of an immiscible Cu-Mo system calculated from first-principles and a derived n-body potential. Phys Rev B 69, 024202 (2004).

[b44] Nishiyama Z. Mechanism of transformation from face-centred to body-centred cubic lattice. Sci Rep Tohoku Imp Univ 23, 637–664 (1934).

[b45] Wasserman G. Arch Eisenhuttenw 16, 647 (1933).

[b46] Kurdjumov G. V. & Sachs G. Z Phys 64, 325 (1930).

[b47] Beyerlein I. J., Wang J., Kang K., Zheng S. J. & Mara N. A. Twinnability of bimetal interfaces in nanostructured composites. Mater Res Lett 1, 89–95 (2013).

[b48] Mitchell T. E., Lu Y. C., Griffin A. J., Nastasi M. & Kung H. Structure and mechanical properties of copper/niobium multilayers. J Am Ceram Soc 80, 1673–1676 (1997).

[b49] Kato M., Wada M., Sato A. & Mori T. Epitaxy of cubic-crystals on (001) cubic substrates - overview no. 78. Acta Metall 37, 749–756 (1989).

[b50] Shimatsu T. & Uomoto M. Atomic diffusion bonding of wafers with thin nanocrystalline metal films. J Vac Sci Technol B 28, 706–714 (2010).

[b51] Dahmen U. Orientation relationships in precipitation systems. Acta Metall 30, 63–73 (1982).

[b52] Beyerlein I. J., Mayeur J. R., Zheng S., Mara N. A., Wang J. & Misra A. Emergence of stable interfaces under extreme plastic deformation. Proc Natl Acad Sci U.S.A. 111, 4386–4390 (2014). 24616514 10.1073/pnas.1319436111 PMC3970524

[b53] Seki A., Seidman D. N., Oh Y. & Foiles S. M. Monte Caro simulations of segregation at [001] twist boundaries in a Pt(Au) alloy—I. Results. Acta Metall Mater 39, 3167–3177 (1991).

[b54] Seki A., Seidman D. N., Oh Y. & Foiles S. M. Monte Carlo simulations of segregation at [001] twist boundaries in a Pt(Au) alloy—II. Discussion. Acta Metall Mater 39, 3179–3185 (1991).

[b55] Christian J. W. Deformation by moving interfaces. Metall Trans A 13, 509–538 (1982).

[b56] Holm E. A., Miodownik M. A. & Rollett A. D. On abnormal subgrain growth and the origin of recrystallization nuclei. Acta Mater 51, 2701–2716 (2003).

[b57] Pond R. C., Ma X. & Hirth J. P. Geometrical and physical models of martensitic transformations in ferrous alloys. J Mater Sci 43, 3881–3888 (2008).

[b58] Rohrer G. S., Holm E. A., Rollett A. D., Foiles S. M., Li J. & Olmsted D. L. Comparing calculated and measured grain boundary energies in nickel. Acta Mater 58, 5063–5069 (2010).

[b59] Peierls R. Size of dislocation. Proc Phys Soc 52, 34–37 (1940).

[b60] Nabarro F. R. N. Dislocations in a simple cubic lattice. Proc Phys Soc 59, 256–272 (1947).

[b61] Foiles S. M. Temperature dependence of grain boundary free energy and elastic constants. Scr Mater 62, 231–234 (2010).

[b62] Chen S. & Chrzan D. C. Continuum theory of dislocations and buckling in graphene. Phys Rev B 84, 214103 (2011).

[b63] Taupin V., Capolungo L., Fressengeas C., Das A. & Upadhyay M. Grain boundary modeling using an elasto-plastic theory of dislocation and disclination fields. J Mech Phys Solids 61, 370–384 (2013).

[b64] Read W. T. & Shockley W. Dislocation models of crystal grain boundaries. Phys Rev 78, 275–289 (1950).

[b65] Marzouk Y. M. & Najm H. N. Dimensionality reduction and polynomial chaos acceleration of Bayesian inference in inverse problems. J Comput Phys 228, 1862–1902 (2009).

