# Supplementary material for: Computational design of patterned interfaces using reduced order models
Source: Sci Rep. 2014 Aug 29;4:6231. doi: 10.1038/srep06231 (PMC4148653; doi:10.1038/srep06231)
Supplement: Supplementary Information — Supplementary Note [file srep06231-s1.pdf]

SUPPLEMENTARY NOTE FOR:

Computational design of patterned interfaces using reduced order models

A. J. Vattré[a, b], N. Abdolrahim[b], K. Kolluri[b], and M. J. Demkowicz[b]

[a]CEA, DAM, DIF, F-91297 Arpajon, France
[b]MIT Department of Materials Science and Engineering, Cambridge MA, 02139

1. Atomistic simulations

Our atomic models are cylinders of radius R_0=27 nm and height $2h$=20 nm with free surfaces in all directions, as shown in Fig. S1. The models consist of an FCC and BCC layer, each of height h. The crystallographic planes at the interface are $\{111\}_{FCC}$ and $\{110\}_{BCC}$. A relative twist of θ, measured with respect to the Nishiyama-Wasserman orientation relation, is imposed on the BCC side of the interface, as shown in Fig. S1.a).

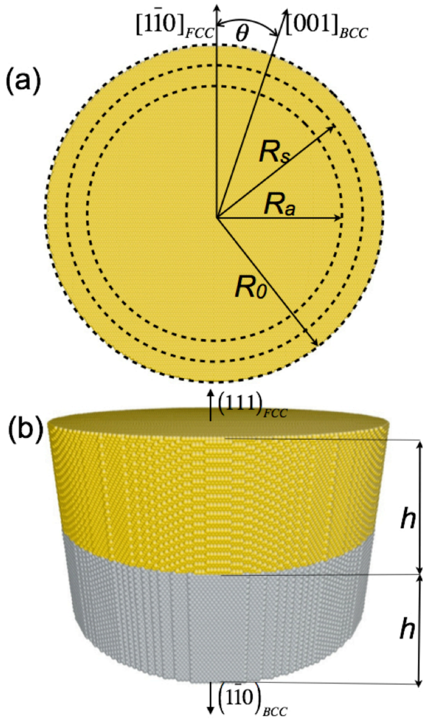

Fig. S1: a) Top and b) side views of one of our atomistic models. FCC atoms are colored yellow while BCC atoms are gray.

To relax these models, neighboring FCC and BCC layers are first translated rigidly with respect to each other in directions parallel to the interface. The lowest energy configuration from such translations is subsequently relaxed by conjugate gradient potential energy minimization (PEM) with multiple restarts using the atomistic modeling code LAMMPS. During PEM, only atoms in an inner cylinder of radius R_s=R_0-2 nm, illustrated in Fig. S1.a), are allowed to move. The positions of atoms in the outer annular region ($R_s \leq r \leq R_0$) are kept fixed to prevent sliding or rotation of the two layers relative to each other. PEM is terminated when the two-norm force on every atom within the inner cylinder is less than 0.1 pN. To avoid edge effects, interface energies and structures are characterized within a region of radius R_a = R_0 -5 nm. Increasing R_0, R_0 -R_s, R_0 -R_a, or h does not change the interface structures or energies reported in this work.

The total interface energy γ is computed as

$$\gamma = \frac{\left(E_f - E_i\right)}{\pi R_a^2} - \gamma_{(111)_{fcc}} - \gamma_{(110)_{bcc}} \, .$$

Here, E_f is the sum of atom energies in the cylinder of radius R_a for a relaxed interface while E_i is the sum of atom energies in the cylinder of radius R_a when the FCC and BCC layers are separated. The free surface energies $\gamma_{(111)_{fcc}}$ and $\gamma_{(110)_{bcc}}$ are computed in a separate calculation. To enable comparison between the ROMM and atomistic calculations, all plots of interface energy as a function of the twist angle θ have been shifted so that the minimum energy occurs at 0 J/m^2. The amounts by which the plots were shifted are given in Table SI. Fig. 4 in the text compares energies from the ROMM and atomistic simulations for Cu/Nb and Ag/V. Fig. S2 below shows similar plots for Cu/Fe and Cu/Mo. Values for *ad hoc* parameters P, Q, and R (Eqn. 4, 5, and 6 in the text) are also plotted.

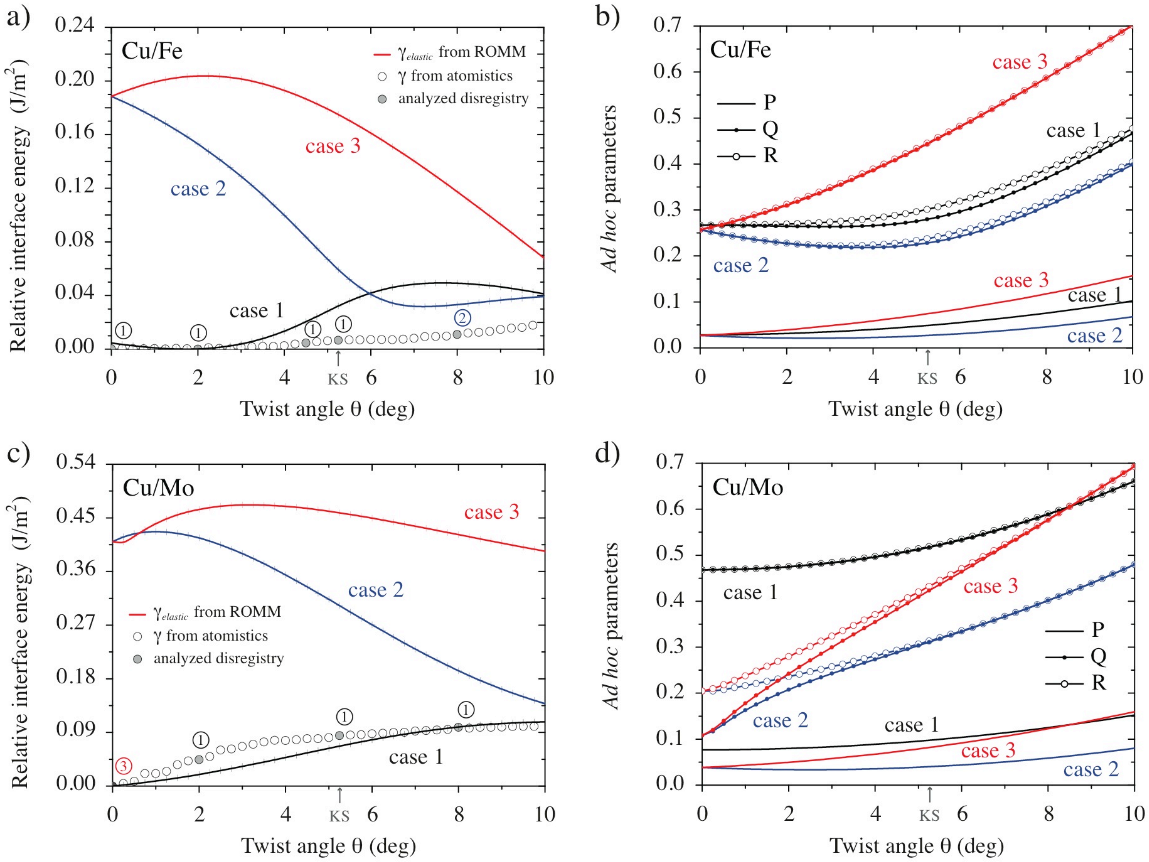

Fig. S2: Interface energies computed as a function of θ using our ROMM and atomistic modeling for a) Cu/Fe and c) Cu/Mo. Filled circles indicate atomic models whose disregistry was analyzed. The ringed numbers next to them state the case that best matches the atomic disregistry. *Ad hoc* parameters P, Q, and R (Eqn. 4, 5, and 6) for b) Cu/Fe and d) Cu/Mo.

Table SI: Amounts by which interface energies were shifted in Fig. 4 and Fig. S2.

Composition	γ, atomistic model (J/m^2)	$\gamma_{elastic}$, ROMM (J/m^2)
Cu/Nb	0.545	0.485
Ag/V	0.847	0.241
Cu/Fe	0.506	0.292
Cu/Mo	1.546	0.416

2. Disregistery analysis

Disregistries in the interfaces studied are superpositions of contributions from two sets of misfit dislocations. To compare with qFBE predictions, we must separate the atomistic disregistry into contributions from each individual set. A single set of parallel misfit dislocations with perfectly compact cores produces either a staircase- or sawtooth-shaped disregistry, depending on the configuration from which displacements are measured. If displacements are measured from the reference state in Fig. 2 (see text) to the natural state after all local interface relaxations have occurred, a staircase disregistry results. A sawtooth disregistry arises if displacements are measured from the unrelaxed natural state created by the mapping in Fig. 2 to the natural state after interface relaxations have occurred. In both approaches, discontinuities in the disregistry correspond to dislocation cores. Either approach may be used to analyze disregistries in atomistic simulations. We adopt the latter and therefore expect to see sawtooth-shaped disregistry plots associated with each set of misfit dislocations. However, since real dislocation cores are not perfectly compact, we expect rapid variations in disregistry—rather than discontinuities—around dislocations cores.

Fig. S3.a) shows an illustrative disregistry plot, computed for a Cu/Nb interface at θ=1°. The disregistry is a position dependent vector field computed by subtracting the relative position of neighboring FCC and BCC atoms in the unrelaxed interface from the relative position between the same two atoms in the relaxed interface. Misfit dislocation Burgers lie in the interface plane for all cases studied here. Thus, we restrict attention to disregistry components in the interface plane only. We compare this disregistry with each of the three cases predicted by the qFBE.

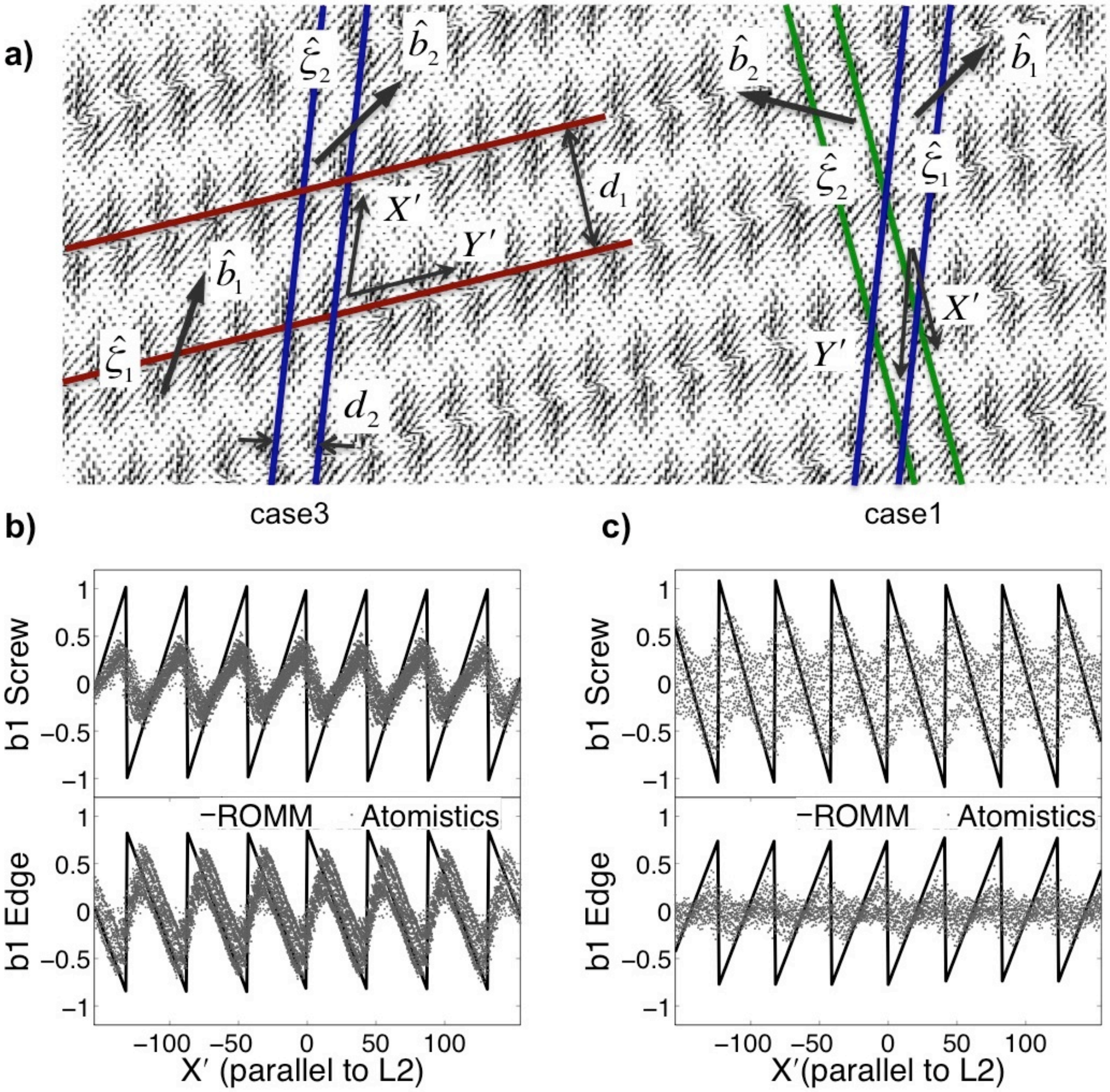

Fig. S3: a) Disregistery vectors of CuNb interface with θ=1°. Dislocation patterns of case 1 and 3 are highlighted and marked with burgers vectors b_i, line directions ζ_i and spacing d_i for each set of dislocations. b) Comparison of the screw and edge disregistery of dislocation set 1 along dislocation set 2 for case 3 according to ROMM and MD results. c) Comparison of the screw and edge disregistery of dislocation set 1 along dislocation set 2 for case 1 according to ROMM and MD results

For example, Fig. S3.a) shows the pattern of misfit dislocations predicted by cases 1 and 3 (see Fig. 3 in text). To isolate the contribution of each dislocation set to the disregistry, we use an oblique coordinate system with axes X' and Y' parallel to the dislocation directions, as in Fig. S3.a). We plot disregistry as a function of its position projected onto one of these axes. This way, all disregistries a given distance along that axis from the core of one set of misfit dislocations are plotted at the same abscissa. For example, any disregistry projected along $\hat{\xi}_2$ plotted to the same abscissa corresponds to the same distance from a set 1 dislocation core.

Therefore, by plotting disregistry as a function of position projected onto $\hat{\xi}_i$ for one set of misfit dislocations in the oblique coordinate system in Fig. S3.a), variations of disregistry due to the other set of misfit dislocations are clearly displayed. In the example above, variations in disregistry due to set 1 may be distinguished by projecting disregistry positions onto $\hat{\xi}_2$ and vice versa. In addition to projecting disregistry positions, we also distinguish between screw and edge components of the disregistry itself. When the disregistry position is projected along $\hat{\xi}_2$, we decompose disregistry into components parallel and perpendicular to $\hat{\xi}_1$, i.e. the screw and edge components for set 1 misfit dislocations.

Fig. S3.b) shows the screw and edge components of disregistry for set 1 as a function of position projected along $\hat{\xi}_2$ for the disregistry in Fig. S3.a). The dislocation line directions correspond to case 3 from the qFBE. A definite sawtooth-shaped pattern is seen, as expected. Discrepancies between the predicted and computed desregistries in the vicinity of the predicted "sawtooth" discontinuities are due to the non-zero width of misfit dislocation cores in the atomistic model. A similar plot may be made for screw and edge disregistries of set 2 as a function of position projected along $\hat{\xi}_1$. The example in Fig. S3.b) compares the simulated atomic disregistry with the case 3 disregistry for a Cu/Nb interface at θ=1°. These two disregistries are in good agreement. Indeed, our ROMM predicts that case 3 has lowest γ_{elastic} for this interface. For contrast, Fig. S3.c) compares atomic disregistry with case 1 for the same interface. The match between the predicted and simulated disregistries is visibly worse for this case.

To assess the discrepancy between predicted and simulated disregistries quantitatively, we compute the root mean square discrepancy (RMSD) between predicted and simulated disregistries:

$$RMSD = \sqrt{\frac{\sum_{t=1}^{k}(y_{AS} - \hat{y}_{qFBE})^2}{k}}.$$

Here, y_{AS} is the disregistery from atomistic simulations for either the screw or edge component along one dislocation line while $\hat{y}_{qFBE}$ is the corresponding desregistry predicted by the qFBE for the case of interest. k is the number of data points. We compute separate RMSD values for both the screw and edge components for both sets of dislocations for the case of interest and add them together. The case with lowest total RMSD is taken as the best match to the simulated disregistry.

For the Cu/Nb interface at θ=1° described above, RMSD is lowest for case 3. This case therefore best describes the misfit dislocation pattern, in agreement with the prediction of our ROMM. Table SII shows comparisons of total RMSD for all three cases for all

interfaces investigated here.

Table SII: Total RMSD values for all the interfaces investigated in this study.

	θ	0° (NW)	1°	2°	4.5°	5.26° (KS)	8°
Cu/Nb	Case1	2.36	2.36	2.39		2.24	2.08
	Case2	2.13	2.18	2.24		2.29	2.39
	Case3	2.12	2.06	2.04		1.91	1.96
Ag/V	Case1	2.31		2.37	2.71	2.41	2.26
	Case2	2.56		2.67	2.55	2.64	2.45
	Case3	2.61		2.78	2.9	2.85	2.52
Cu/Fe	Case1	1.65		1.77	1.81	1.87	1.8
	Case2	2.16		2.18	1.95	2.09	1.75
	Case3	2.01		1.96	1.96	2	2.1
Cu/Mo	Case1	2.43		2.32		2.03	1.81
	Case2	2.27		2.45		2.23	2.37
	Case3	2.25		2.45		2.10	2.17
